# From coverage to care: how basic medical insurance shapes patients’ experiences of primary care in China

**DOI:** 10.3389/fepid.2026.1785586

**Published:** 2026-06-02

**Authors:** Chufeng Xu, Wenhu Xu, Jiamin You, Gong Chen, Shuhan Miao

**Affiliations:** 1 Institute of Population Research, Peking University, Beijing, China; 2 School of Humanities and Law, University of Science and Technology Beijing, Beijing, China

**Keywords:** healthcare services, hierarchical medical system, medical insurance, middle-aged and older adults, primary care

## Abstract

**Background:**

With the intensifying trend of population aging in China, the number of middle-aged and older adults continues to rise, and their demand for medical services is also increasing. Middle-aged and older adults can benefit from the convenience and protection provided by the hierarchical diagnosis and treatment system through medical insurance policies.

**Objectives:**

This study draws on Andersen's health service utilization model to examine how basic medical insurance shapes healthcare experiences among middle-aged and older adults with a primary-care orientation under China's hierarchical diagnosis and treatment system.

**Methods:**

This study merges data from the 2016 and 2018 Chinese Family Panel Studies (CFPS) to create a balanced panel, with a total sample size of 6,114. This study uses fixed-effects models and panel ordered Logit models to examine the association between basic health insurance and satisfaction with healthcare facility conditions and healthcare service levels. Additionally, propensity score matching was used within the primary-care-oriented sample to improve comparability between insured and uninsured respondents, followed by fixed-effects estimation as a robustness check.

**Results:**

Descriptive analysis shows that, compared with 2016, the medical expenditure rate in 2018 decreased by 1.16%, while the hospitalization rate and the proportion of doctor visits increased modestly. The two-way fixed-effects results suggest that basic health insurance is positively associated with satisfaction with healthcare facility conditions, corresponding to an average within-person increase of 0.112 points on the observed 1–5 evaluation scale. By contrast, no statistically significant association is found between basic health insurance and healthcare service level. The ordered-model results and the PSM-based robustness checks are broadly consistent with the benchmark estimates. In addition, the strength of the association varies across subgroups defined by health status, age, gender, region, and household registration.

**Conclusions:**

Basic health insurance is associated with more favorable evaluations of healthcare facility conditions among middle-aged and older adults with a primary-care orientation, whereas its association with healthcare service level is not statistically robust. These findings suggest that the relationship between insurance coverage and patient-reported primary care experience may differ across dimensions of healthcare evaluation and across population subgroups.

**Contributions:**

This study has important practical significance for promoting the hierarchical diagnosis and treatment system, optimizing the allocation of medical resources, and alleviating the problem of medical order. Through in-depth research and exploration of relevant issues, it can provide useful references for policy makers and bring more convenient and efficient medical services to patients.

## 1 Introduction

In the context of China's rapidly aging population, the demand for healthcare services among middle-aged and older adults in primary communities continues to rise. To date, the medical service system in China exhibits an “inverted triangle” pattern, with high-quality medical resources concentrated in large hospitals, while primary healthcare services remain relatively underdeveloped. This issue has not yet been effectively addressed. According to data recorded in the China Statistical Yearbook and the China Health and Family Planning Statistical Yearbook (1), the total number of medical visits has shown a general upward trend after years of hierarchical medical reform. From 343.892 million visits in 2017, the number increased to 357.738 million in 2018, and further rose to 388.38 million visits in 2021 (2). However, the number of visits to primary healthcare institutions was 442.892 million in 2017, which decreased to 440.632 million in 2018, and further dropped to 425.024 million in 2021 after the COVID-19 pandemic. Regarding hospital bed turnover rates and utilization, the total number of medical visits to hospitals decreased from 32.3 times per bed (with a bed occupancy rate of 79.7%) in 2017, to 32.2 times per bed (with an occupancy rate of 84.2%) in 2018, and then to 28.5 times per bed (with an occupancy rate of 74.6%) in 2021. Meanwhile, for primary healthcare institutions, the number of bed turnovers dropped from 31.2 times per bed (with a bed occupancy rate of 60.3%) in 2017 to 29.6 times per bed (with an occupancy rate of 58.4%) in 2018, and further decreased to 23.1 times per bed (with an occupancy rate of 47.4%) in 2021 (3). This indicates that the “difficulty in seeing a doctor” remains a prominent and severe issue (4, 5), both in terms of medical visits and hospitalizations (6). The “inverted triangle” medical structure suggests that the overcrowding in higher-level hospitals, represented by tertiary hospitals, remains unresolved. Consequently, the evaluation of healthcare services, as shaped by patients' experiences of difficulty in accessing healthcare, deserves greater attention. The utilization and evaluation of primary healthcare services should remain a focus.

Many scholars primarily focus on the factors influencing the evaluation of healthcare services. In studies from Europe, scholars (7–9) have noted that the accessibility of medical resources, like health insurance, is a key factor affecting the satisfaction of middle-aged and older patients. Other scholars (10–12) have found that high-level physicians, apart from health insurance, are a significant determinant of patient satisfaction with medical services. Health insurance, being the most common form of medical resource, plays an indispensable role in healthcare service evaluation. For example, Taiwanese scholars (13, 14) analyzed data from Taiwan and concluded that health insurance policies have a significant impact on public satisfaction with healthcare. It is evident that these scholars focus on identifying factors affecting healthcare service satisfaction, often using multiple explanatory variables to explain the macro situation. However, they lack a concrete understanding of the healthcare situation in mainland China, and fail to account for the interaction between variables.

Chinese mainland scholars have conducted extensive research on the relationship between domestic health insurance policies and healthcare service evaluation. Some studies (15) using comparative analysis across eight cities showed that health insurance policies in different regions have substantial effects on residents' satisfaction with healthcare services. The current allocation of urban and rural healthcare resources remains unequal (16), with urban residents enjoying significantly better health insurance benefits than rural residents. Furthermore, the influence of individual characteristics on satisfaction is stronger for urban residents than for rural residents (17). And current research also indicates that patients' overall satisfaction with healthcare services is significantly impacted by their trust in medical services and attitudes toward health policies. This finding provides valuable insights for improving healthcare services in China (18). The unequal distribution of medical resources, particularly the disparity in health insurance benefits between urban and rural residents, has been a focal point of China's healthcare reform. Studies show that middle-aged and older adults with health insurance are more likely to use outpatient services compared to those without insurance, and those with insurance have lower out-of-pocket medical expenses in Zhejiang Province, whereas the situation is reversed in Gansu Province (19). For urban residents, individual characteristics such as gender, education level, and insurance status are significantly correlated with satisfaction with healthcare services. Additionally, research has identified high medical costs as a primary reason for patient dissatisfaction (20).

Research on the relationship between health insurance and healthcare services under hierarchical diagnosis and treatment reform cannot be separated from the perspective of various institutional studies (21). Hierarchical diagnosis and treatment is a key element in China's new healthcare reform (22). The impact of China's new healthcare reform on health insurance coverage and the efficiency of medical service utilization is complex. Moreover, the increase in health insurance coverage has not significantly improved the overall efficiency of healthcare service utilization and may even lead to a decrease in system efficiency (23, 24). The ongoing hierarchical diagnosis and treatment reforms in China, are essential measures to enhance patient satisfaction. Between 2010 and 2016, China made significant progress in improving access to medical services and financial protection, but this has not been accompanied by improvements in satisfaction with the healthcare system. Despite increased health insurance coverage (22), patients are more likely to seek medical services in hospitals rather than in primary medical institutions, indicating the need to further strengthen the hierarchical diagnosis and treatment system (25–27). Among these efforts, the pricing mechanism is crucial. Only when patients accurately perceive the price differences between hospitals of different levels will they be able to appropriately choose healthcare institutions, thereby promoting the implementation of hierarchical diagnosis and treatment. On the other hand, as the hierarchical diagnosis and treatment system gradually takes effect, an improved primary healthcare service system will change patients' perceptions and further drive improvements in health insurance policies (28–30), ultimately enhancing patient satisfaction with medical services. Under the new healthcare reform, the job satisfaction of rural community doctors has been significantly impacted. Policymakers should pay more attention to improving the primary medical environment and reward mechanisms to ensure the stability and sustainability of the hierarchical diagnosis and treatment system (31, 32).

Some scholars have analyzed data from urban populations and concluded that residents' satisfaction with healthcare services increased after the implementation of healthcare reform, while others have pointed out that hierarchical diagnosis and treatment has altered the previous medical service order and customs, particularly in rural areas, where the effects have been the opposite (33, 34). The universal health insurance policy replaced the previous “three-free” policy (35). Although the standards for outpatient and inpatient care have improved, residents' perceived satisfaction has declined. Some scholars have also analyzed the 2011 and 2013 China Health and Retirement Longitudinal Study data, finding that health insurance has not reduced individuals' medical costs. In some cases (36), patients may even have to pay more for outpatient services. Moreover, this research found that although health insurance increased inpatient service utilization and reduced out-of-pocket expenditures for hospitalization, its protective effect on outpatient medical expenses was weak, reflecting inequalities and coverage gaps in the health insurance system (37, 38). Existing research on health insurance and healthcare service evaluation has made significant progress in terms of research focus, dimensions, and methods. However, few studies have conducted a comprehensive analysis of the relationship between healthcare service evaluation and basic health insurance for middle-aged and older adults in the context of hierarchical diagnosis and treatment, and even fewer have conducted comparative analyses before and after the implementation of the new healthcare reforms.

Andersen's health service utilization model, first proposed in 1968, has undergone several stages of development and refinement. Early versions of the model emphasized individual predisposition, available resources, and health needs, while later refinements incorporated features of the healthcare system and clarified the relationships among determinants of healthcare use. In its mature form, the model explains healthcare utilization through four broad dimensions: predisposing factors, enabling factors, need factors, and health behavior factors. Within this framework, basic medical insurance is conceptualized in this study as a key enabling factor, because it may reduce financial barriers, improve affordability, and shape access to primary healthcare services. In the context of China's hierarchical diagnosis and treatment system, this enabling role may be particularly important for middle-aged and older adults with a primary-care orientation, whose healthcare experiences are likely to depend not only on need factors, but also on whether institutional resources make primary care more accessible and usable. Guided by this framework, the present study specifies basic medical insurance as the focal enabling variable and examines whether this enabling resource is associated with more favorable patient-reported evaluations of primary care, net of predisposing factors, need-related conditions, and health behavior characteristics. This study adopts the fourth-stage framework of the model to examine how basic medical insurance is associated with primary healthcare experiences among middle-aged and older adults, especially how to respond to policy adjustments and social changes by changing the utilization behavior and patterns of medical services, thereby providing empirical evidence relevant to health policy design. Compared with prior studies, this study makes three contributions. First, it focuses specifically on middle-aged and older adults with a primary-care orientation, a population group that has received limited attention in the literature on China's hierarchical diagnosis and treatment system. Second, rather than examining healthcare utilization alone, it emphasizes patient-reported evaluations of primary care, including healthcare facility conditions and healthcare service level. Third, it combines fixed-effects models, ordered response models, and PSM-based robustness checks to examine the association between basic health insurance and primary care experience while addressing both observable selection and unobservable time-invariant heterogeneity.

## 2 Materials and methods

**Figure 1:**
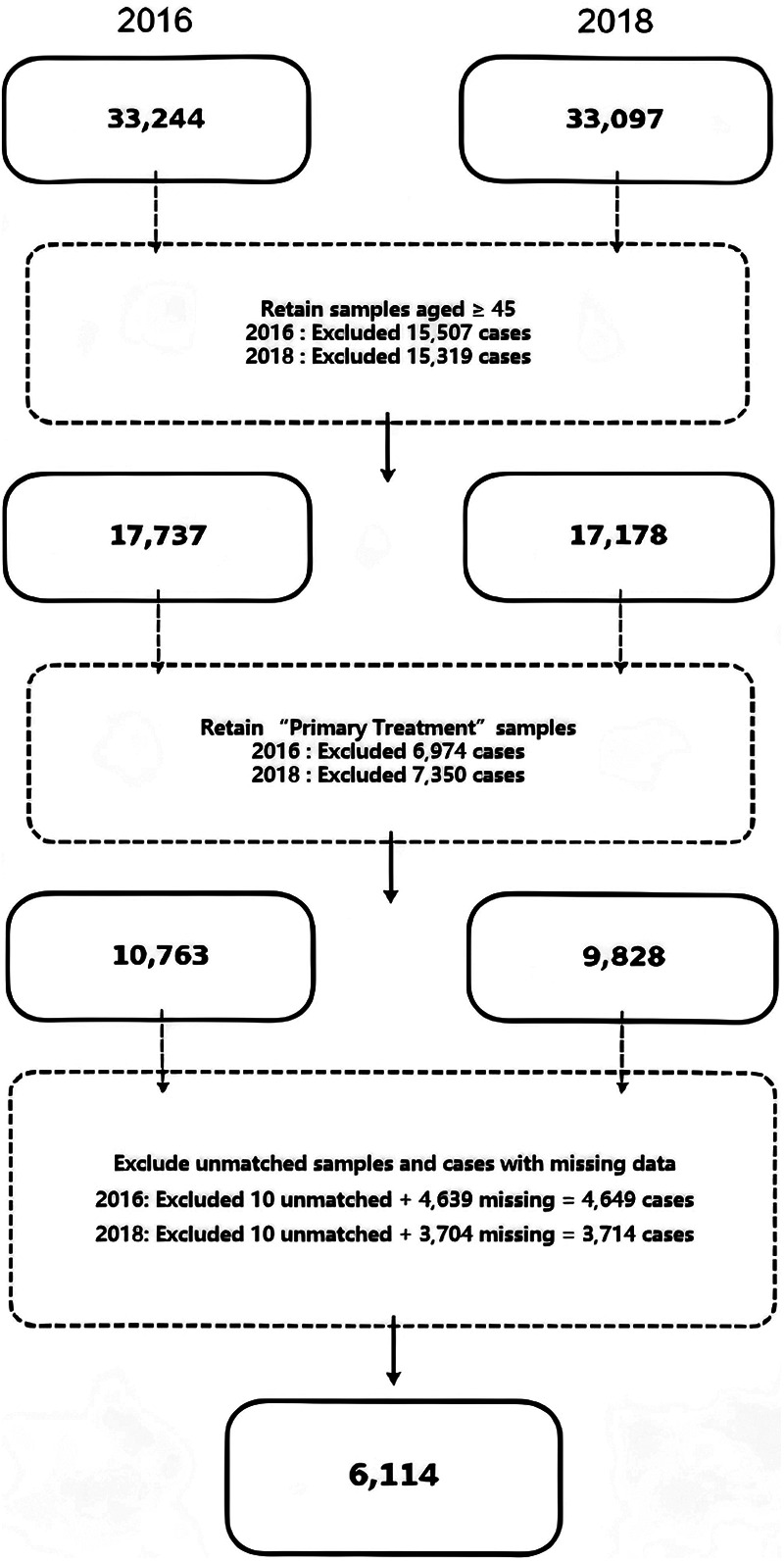
Flowchart of sample selection and balanced panel construction.

The data for this study are derived from the China Family Panel Studies (CFPS) (39, 40), which initiated its baseline survey in 2010 and covers 25 provinces and cities across China. The database reflects the economic, social, and population health conditions of China, providing valuable data support for analyzing the hierarchical diagnosis and treatment policy. Based on the medical reform targets outlined in the “Opinions” for the year 2017, this study selected data from 2016 to 2018, focusing specifically on individuals aged 45 and older. As shown in Figure 1, the sample selection followed a stepwise procedure. The initial sample included 33,244 individuals in 2016 and 33,097 individuals in 2018. First, the sample was restricted to respondents aged 45 years and above, which led to the exclusion of 15,507 cases in 2016 and 15,919 cases in 2018, leaving 17,737 and 17,178 observations, respectively. Second, based on the CFPS item “Where do you seek medical care?” (QP601), respondents whose usual source of care was a primary-level medical institution were identified as having a primary-care orientation (“primary treatment”). Applying this criterion excluded a further 6,974 cases in 2016 and 7,350 cases in 2018, resulting in 10,763 and 9,828 observations, respectively. Third, cases with missing values on key variables and cases with unmatched IDs across waves were excluded, and the sample was further restricted to respondents enrolled in only one type of basic health insurance. This final step excluded 4,649 cases in 2016 and 3,714 cases in 2018. After matching respondents observed in both waves to construct a balanced panel, the final analytical sample consisted of 6,114 individuals. Only respondents who met the primary-care-orientation criterion and could be consistently matched across both waves were retained in the final balanced panel. Respondents whose QP601 status changed across waves were excluded from the final balanced panel.

### 2.1 Independent variables

China has achieved near-universal basic medical insurance coverage, but coverage is not complete in a strict empirical sense. Official sources have described the national basic medical insurance coverage rate as remaining stable at around 95%, China's basic medical insurance system mainly included Urban Employee Basic Medical Insurance (UEBMI) and the resident-side schemes, namely Urban Resident Basic Medical Insurance (URBMI) and the New Rural Cooperative Medical Scheme (NRCMS). In January 2016, the State Council issued the policy to integrate URBMI and NRCMS into a unified Urban–Rural Resident Basic Medical Insurance (URRBMI) system, and this integration was being implemented during our study period.

To capture the association of a single insurance status with healthcare evaluation, this study retained only respondents enrolled in one type of basic medical insurance. The core explanatory variable is whether the respondent was enrolled in basic medical insurance. In this study, the “no-coverage” group refers to respondents who reported not being enrolled in any basic medical insurance scheme at the time of the survey; it does not represent a separate insurance category.

### 2.2 Dependent variables

Healthcare service evaluation: The first dependent variable was satisfaction with healthcare facility conditions, measured using CFPS item QP602. The second dependent variable was satisfaction with healthcare service level, measured using CFPS item QP603. Both variables were coded on an ordered five-category scale from 1 to 5, where 1 represents the lowest level of evaluation and 5 represents the highest level of evaluation.

Given the ordered nature of these outcomes, the main analysis placed greater emphasis on ordered response models for substantive interpretation. At the same time, linear fixed-effects models were retained as a supplementary specification to facilitate comparison with prior panel studies and to absorb time-invariant unobserved heterogeneity at the individual level. Accordingly, coefficients from the linear fixed-effects models should be interpreted as average within-person changes in the observed satisfaction score, rather than as percentage changes in satisfaction.

### 2.3 Control variables

Following Andersen's framework, basic medical insurance is treated in this study as an enabling factor that may shape respondents' access to and evaluations of primary healthcare services. This study referred to the Andersen model in previous research, and after conducting univariate analysis of variance, selected propensity factor variables with statistical significance lower than 5% (e.g., age, gender, marital status, party membership status), enabling factors (e.g., post-tax monthly income, education level), need factors (e.g., presence of chronic diseases, self-rated health), and health behavior variables (e.g., smoking and drinking habits), and included these variables in the model for control. Prior to constructing the regression model, this study conducted a detailed check for potential multicollinearity among variables. The variance inflation factors (VIF) for the selected variables were all less than 2, well below the conventional threshold of 10, ensuring that multicollinearity was not present in the model. This classification aligns the empirical specification with Andersen's behavioral model, allowing the estimated association for basic medical insurance to be interpreted specifically as the effect of an enabling resource net of predisposing, need, and health behavior factors.

### 2.4 Statistical analysis

In the healthcare service evaluation section, this study used two indicators from the CFPS database—QP602 (satisfaction with healthcare facility conditions) and QP603 (satisfaction with healthcare service level)—as the dependent variables. Therefore, their ordinal nature was explicitly taken into account in model specification and interpretation.

Linear fixed-effects and two-way fixed-effects models were estimated as baseline specifications. These models treated the observed 1–5 score as an approximately continuous outcome and were used primarily to examine the direction, statistical significance, and within-individual association between basic health insurance and healthcare evaluations over time. In this setting, regression coefficients suggest changes in the observed satisfaction score, not percentage changes and not marginal probabilities. Because the dependent variables are ordinal rather than truly continuous, panel ordered Logit fixed-effects models were additionally estimated as a complementary specification for robustness and interpretation. Odds ratios are reported to facilitate interpretation of shifts toward more favorable evaluation categories.

As a robustness check, propensity score matching (PSM) was further combined with fixed-effects estimation. In the PSM procedure, the treatment group was defined as respondents enrolled in basic health insurance, and the control group consisted of comparable respondents without insurance within the primary-care-oriented sample. The propensity score model included the same observable covariates as the benchmark regressions, and balance was assessed after matching within the region of common support. The combination of PSM and fixed-effects estimation serves different purposes. PSM is used to improve comparability between insured and uninsured respondents on observed covariates, thereby reducing observable selection bias. Fixed-effects models, in contrast, absorb unobserved individual characteristics that remain constant over time, such as stable health preferences or long-standing attitudes toward healthcare utilization. Combining the two approaches therefore provides a more transparent robustness strategy by addressing observable selection and unobservable time-invariant heterogeneity simultaneously. All analyses were conducted using Stata 17.0, with statistical significance defined as *α* = 0.05. Given the observational nature of the data, all estimated coefficients are interpreted as associations rather than causal effects.

## 3 Results

### 3.1 Descriptive analysis

As shown in Table 1, the medical expenditure rate in 2016 was 75.27%, with a health insurance coverage rate of 94.75%. The mean proportion of out-of-pocket medical expenses was 65.0% (±43.5%), the mean total medical expenses (logarithmic scale) was 4.902 (±3.104), and the mean out-of-pocket medical expenses (logarithmic scale) was 4.662 (±3.051). The proportion of individuals rating the healthcare service level as “excellent” was 8.59%, and the proportion of individuals expressing “very satisfied” with the healthcare facility conditions was 4.86%. In the past 12 months, the hospitalization rate was 9.75%, while the proportion of individuals who had seen a doctor was 26.23%.

**Table 1:** Variable meaning, assignment, and basic information.

Variable	Value	2016 (*N* = 6,114)		2018 (*N* = 6,114)		Panel data (*N* = 12,228)	
		Frequency	Proportion	Frequency	Proportion	Frequency	Proportion
Healthcare service level							
Very poor	1	64	1.05%	681	11.14%	745	6.09%
Poor	2	342	5.59%	2,472	40.43%	2,814	23.01%
Average	3	3,528	57.70%	2,143	35.05%	5,671	46.38%
Good	4	1,655	27.07%	681	11.14%	2,336	19.10%
Excellent	5	525	8.59%	137	2.24%	662	5.41%
Satisfaction with healthcare facility conditions							
Very dissatisfied	1	34	0.56%	619	10.12%	653	5.34%
Dissatisfied	2	359	5.87%	3,642	59.57%	4,001	32.72%
Average	3	2,174	35.56%	1,262	20.64%	3,436	28.10%
Satisfied	4	3,250	53.16%	502	8.21%	3,752	30.68%
Very satisfied	5	297	4.86%	89	1.46%	386	3.16%
Health insurance coverage							
No	0	321	5.25%	345	5.64%	666	5.45%
Yes	1	5,793	94.75%	5,769	94.36%	11,562	94.55%
Age							
44∼59 years	0	3,609	59.03%	3,227	52.78%	6,836	55.90%
60∼69 years	1	1,827	29.88%	1,956	31.99%	3,783	30.94%
70∼79 years	2	605	9.90%	812	13.28%	1,417	11.59%
80 + years	3	73	1.19%	119	1.95%	192	1.57%
Gender							
Female	0	3,026	49.49%	3,026	49.49%	6,052	49.49%
Male	1	3,088	50.51%	3,088	50.51%	6,176	50.51%
Party membership							
No	0	5,651	92.43%	6,105	99.85%	11,756	96.14%
Yes	1	463	7.57%	9	0.15%	472	3.86%
Marital status							
Single	1	65	1.06%	66	1.08%	131	1.07%
Married (with spouse) or cohabiting	2	5,472	89.50%	5,370	87.83%	10,842	88.67%
Divorced or widowed	3	577	9.44%	678	11.09%	1,255	10.26%
Education level							
Illiterate/semiliterate	1	2,527	41.33%	2,422	39.61%	4,949	40.47%
Primary school	2	1,575	25.76%	1,668	27.28%	3,243	26.52%
Junior high school	3	1,447	23.67%	1,455	23.80%	2,902	23.73%
Highschool/vocational/technical school	4	510	8.34%	514	8.41%	1,024	8.37%
College or above	5	55	0.90%	55	0.90%	110	0.90%
Household registration status							
Non-agricultural household	0	817	13.36%	795	13.00%	1,612	13.18%
Agricultural household	1	5,297	86.64%	5,319	87.00%	10,616	86.82%
Smoking status							
No	0	4,213	68.91%	4,134	67.62%	8,347	68.26%
Yes	1	1,901	31.09%	1,980	32.38%	3,881	31.74%
Alcohol consumption status							
No	0	4,969	81.27%	4,932	80.67%	9,901	80.97%
Yes	1	1,145	18.73%	1,182	19.33%	2,327	19.03%
Monthly after-tax income and pension							
0 to 1,000 RMB	1	5,873	96.06%	4,285	70.09%	10,158	83.07%
1,001-2,000 RMB	2	113	1.85%	897	14.67%	1,010	8.26%
Above 2,000 RMB	3	128	2.09%	932	15.24%	1,060	8.67%
Self-rated health							
Unhealthy	1	1,124	18.38%	1,286	21.03%	2,410	19.71%
Average	2	1,354	22.15%	980	16.03%	2,334	19.09%
Fairly healthy	3	2,106	34.45%	2,380	38.93%	4,486	36.69%
Healthy	4	876	14.33%	697	11.40%	1,573	12.86%
Very healthy	5	654	10.70%	771	12.61%	1,425	11.65%
Chronic disease in the last six month							
No	0	4,946	80.90%	4,897	80.09%	9,843	80.50%
Yes	1	1,168	19.10%	1,217	19.91%	2,385	19.50%
Hospitalization in the last 12 months							
No	0	5,518	90.25%	5,407	88.44%	10,925	89.34%
Yes	1	596	9.75%	707	11.56%	1,303	10.66%
Consulted a doctor							
No	0	4,510	73.77%	4,341	71.00%	8,851	72.38%
Yes	1	1,604	26.23%	1,773	29.00%	3,377	27.62%
Total sample size		6,114	100%	6,114	100%	12,228	100%

In 2018, the medical expenditure rate decreased slightly to 74.11%, while the health insurance coverage rate was 94.36%. The mean proportion of out-of-pocket medical expenses decreased to 62.9% (±43.8%). The mean total medical expenses (logarithmic scale) increased to 5.011 (±3.246), while the mean out-of-pocket medical expenses (logarithmic scale) increased to 4.759 (±3.179). The proportion of individuals rating the healthcare service level as “excellent” dropped to 2.24%, and the proportion of individuals expressing “very satisfied” with the healthcare facility conditions fell to 1.46%. The hospitalization rate in the past 12 months rose to 11.56%, while the proportion of individuals who had seen a doctor increased to 29.00%.

Comparing 2018 to 2016, the medical expenditure rate decreased by 1.16%. Health insurance coverage dropped by 0.39%. The proportion of out-of-pocket medical expenses decreased by 2.1%. The total medical expenses (logarithmic scale) increased by 0.109. The out-of-pocket medical expenses (logarithmic scale) increased by 0.097. The proportion of individuals rating the healthcare service level as “excellent” decreased by 6.35%. The proportion of individuals who were “very satisfied” with the healthcare facility conditions decreased by 3.40%. The hospitalization rate (inpatient services) increased by 1.81%. The proportion of individuals who had visited a doctor (outpatient services) increased by 2.77%.

The panel data suggest that the overall medical expenditure rate over the two years was 74.69%, with a health insurance coverage rate of 94.55%. The mean proportion of out-of-pocket medical expenses was 64.0% (±43.7%), the mean total medical expenses (logarithmic scale) was 4.957 (±3.176), and the mean out-of-pocket medical expenses (logarithmic scale) was 4.711 (±3.116). The proportion of individuals rating the healthcare service level as “excellent” was 5.41%, and the proportion of individuals expressing “very satisfied” with the healthcare facility conditions was 3.16%. The hospitalization rate over the past 12 months (inpatient services) was 10.66%, while the proportion of individuals who had seen a doctor was 27.62%. The basic statistics for other variables are shown in Table 1. The balanced panel contains 6,114 individuals observed in two waves, yielding 12,228 person-wave observations.

### 3.2 Association between basic health insurance and healthcare service evaluation

Based on prior literature, this study selected satisfaction with healthcare facility conditions and healthcare service level as indicators for evaluating primary healthcare services. Initially, fixed effects models were used for regression analysis. Before conducting the regression, both fixed effects and random effects models were calculated. The Hausman test yielded a result less than 0.001, leading to the use of fixed effects models and two-way fixed effects models for regression analysis. Subsequently, a panel ordered Logit fixed effects model was used to further compare the data.

#### 3.2.1 Fixed effects and two-way fixed effects model results

As shown in Table 2, both the fixed effects model and the two-way fixed effects model show that basic health insurance is significantly associated with higher satisfaction with healthcare facility conditions at the 1% level. In the fixed-effects model, the coefficient of basic health insurance is 0.118, while in the two-way fixed-effects model it is 0.112. Given that the dependent variable is measured on an ordered 1–5 scale, these estimates show that health insurance participation is associated with a modest increase in the reported satisfaction score for healthcare facility conditions.

**Table 2:** Fixed-effects estimates of the association between basic health insurance and healthcare service evaluation among middle-aged and older adults with a primary-care orientation.

Variable	Satisfaction with healthcare facility conditions		Healthcare service level	
	(1)	(2)	(3)	(4)
Basic health insurance	0.118^***^	0.112^***^	0.065	0.059
	(0.042)	(0.042)	(0.044)	(0.044)
Age	−0.050	0.052	−0.070^*^	0.017
	(0.036)	(0.038)	(0.038)	(0.040)
Gender	1.490^**^	1.483^**^	1.476^**^	1.470^**^
	(0.663)	(0.659)	(0.698)	(0.696)
Party membership	0.070	−0.006	0.033	−0.032
	(0.044)	(0.045)	(0.046)	(0.047)
Marital status	0.011	0.035	−0.009	0.011
	(0.026)	(0.026)	(0.027)	(0.027)
Education level	−0.035	0.031	−0.109	−0.054
	(0.072)	(0.072)	(0.076)	(0.076)
After-tax monthly income	−0.075^***^	−0.010	−0.046^***^	0.009
	(0.015)	(0.016)	(0.016)	(0.017)
Smoking status	0.009	0.024	0.026	0.039
	(0.048)	(0.047)	(0.050)	(0.050)
Alcohol consumption status	−0.038	−0.030	0.016	0.022
	(0.035)	(0.034)	(0.036)	(0.036)
Self-rated health	−0.060^***^	−0.057^***^	−0.072^***^	−0.069^***^
	(0.010)	(0.010)	(0.010)	(0.010)
Chronic disease	−0.063^**^	−0.062^**^	−0.079^***^	−0.078^***^
	(0.026)	(0.026)	(0.027)	(0.027)
Hospitalization in the last year	−0.084^***^	−0.073^**^	−0.101^***^	−0.092^***^
	(0.032)	(0.032)	(0.034)	(0.034)
Consulted a doctor	−0.016	−0.007	0.005	0.012
	(0.023)	(0.023)	(0.024)	(0.024)
Control for individual effects	Yes	Yes	Yes	Yes
Control for time effects	No	Yes	No	Yes
Constant	1.874^***^	1.605^***^	2.327^***^	2.098^***^
	(0.374)	(0.373)	(0.394)	(0.394)

Note: Heteroskedasticity-robust standard errors are reported in parentheses; ***p < 0.01, **p < 0.05, *p < 0.1.

By contrast, basic health insurance does not show a statistically significant association with satisfaction with healthcare service level in either model. Among the control variables, self-rated health status, chronic disease, and hospitalization in the past year are consistently negatively associated with satisfaction with healthcare facility conditions. These results suggest that poorer health conditions and recent hospitalization experiences are linked to less favorable evaluations of primary healthcare facilities.

Overall, the fixed-effects results suggest that, among middle-aged and older adults with a preference for primary care, participation in basic health insurance is positively associated with evaluations of healthcare facility conditions, whereas its association with perceived healthcare service level remains statistically insignificant.

#### 3.2.2 Panel ordered logit fixed-effects model results

To further account for the ordinal nature of the dependent variables, this study additionally estimated panel ordered Logit fixed-effects models. As shown in Table 3, the results are generally consistent with those reported in the linear fixed-effects models.

**Table 3:** Odds ratios from panel ordered logit fixed-effects models of the association between basic health insurance and healthcare service evaluation among middle-aged and older adults with a primary-care orientation.

Variable	Satisfaction with healthcare facility conditions		Healthcare service level	
	(1)	(2)	(3)	(4)
Basic health insurance	1.381^**^	1.359^**^	1.200	1.186
Control variable	Yes	Yes	Yes	Yes
Control for individual effects	Yes	Yes	Yes	Yes
Control for time effects	No	Yes	No	Yes
	(0.374)	(0.373)	(0.394)	(0.394)

Odds ratios (ORs) are reported. OR values greater than 1 show higher odds of reporting more favorable evaluations.

Note: Heteroskedasticity-robust standard errors are reported in parentheses; ***p < 0.01, **p < 0.05, *p < 0.1.

For satisfaction with healthcare facility conditions, basic health insurance is positively and significantly associated with higher evaluations in both specifications. In Model (1), the estimated odds ratio is 1.381 (*p* < 0.05). After further controlling for time fixed effects in Model (2), the odds ratio remains positive and statistically significant at 1.359 (*p* < 0.05). These results show that enrollment in basic health insurance is associated with higher odds of reporting more favorable evaluations of healthcare facility conditions among middle-aged and older adults with a primary-care orientation.

By contrast, for healthcare service level, the estimated odds ratios of basic health insurance remain positive in both Model (3) and Model (4), but neither reaches statistical significance. This suggests that the association between basic health insurance and the evaluation of healthcare service level is not robust in the ordered fixed-effects specifications.

Overall, the panel ordered Logit fixed-effects results reinforce the main conclusion of this study: basic health insurance is more clearly associated with improved evaluations of healthcare facility conditions than with evaluations of healthcare service level among middle-aged and older adults with a primary-care orientation.

### 3.3 Robustness check

As shown in Table 4, the propensity score matching fixed-effects regression (PSM-FEM) results further support the robustness of the benchmark findings by improving covariate balance between insured and uninsured respondents on observables while continuing to control for time-invariant unobserved individual heterogeneity. After matching, the estimated coefficient of basic health insurance on satisfaction with healthcare facility conditions remains consistently positive and statistically significant across all matching strategies, including nearest-neighbor matching (K = 4), caliper matching (Cal = 0.05), caliper matching (Cal = 0.01), and kernel matching. In all four specifications, the estimated coefficient is 0.112 and remains significant at the 1% level. This suggests that the positive association between enrollment in basic health insurance and satisfaction with healthcare facility conditions among middle-aged and older adults with a primary-care orientation is robust to alternative matching procedures. Balance diagnostics suggested substantial improvement in covariate balance after matching, with all standardized mean differences reduced to acceptable levels within the region of common support.

**Table 4:** Robustness checks based on propensity score matching and fixed-effects regression among middle-aged and older adults with a primary-care orientation.

Variable name	Satisfaction with healthcare facility conditions				Healthcare service level			
	PSM-FEM Nearest neighbor matching (K = 4)	PSM-FEM Caliper matching (Cal = 0.05)	PSM-FEM Caliper matching (Cal = 0.01)	PSM-FEM Kernel matching	PSM-FEM Nearest neighbor matching (K = 4)	PSM-FEM Caliper matching (Cal = 0.05)	PSM-FEM Caliper matching (Cal = 0.01)	PSM-FEM Kernel matching
Basic health insurance	0.112***	0.112***	0.112***	0.112***	0.060	0.060	0.060	0.060
	(0.042)	(0.042)	(0.042)	(0.042)	(0.044)	(0.044)	(0.044)	(0.044)
Control variables	Control	Control	Control	Control	Control	Control	Control	Control
Control for individual effects	Yes	Yes	Yes	Yes	Yes	Yes	Yes	Yes
Control for time effects	Yes	Yes	Yes	Yes	Yes	Yes	Yes	Yes
Constant	1.604***	1.604***	1.604***	1.604***	2.084***	2.084***	2.084***	2.084***
	(0.372)	(0.372)	(0.372)	(0.372)	(0.394)	(0.394)	(0.394)	(0.394)

By contrast, for healthcare service level, the estimated coefficient of basic health insurance is 0.060 across all matching specifications, but none of these estimates is statistically significant. This suggests that, although the direction of the association remains positive, the relationship between basic health insurance and the evaluation of healthcare service level is not sufficiently robust in the matched sample.

Overall, the PSM-FEM results show that the main finding of this study is stable: basic health insurance is robustly associated with higher satisfaction with healthcare facility conditions, but not with healthcare service level, among middle-aged and older adults with a primary-care orientation.

### 3.4 Heterogeneity in the association between basic health insurance and healthcare service evaluation

The heterogeneity analysis (Table 5) suggests that the association between basic health insurance and healthcare service evaluation varies across subgroups, although the pattern is not uniform across all dimensions. In terms of health status, the positive association between basic health insurance and healthcare service evaluation is more evident among middle-aged and older adults with chronic diseases, particularly in their assessment of healthcare service level. Age-related differences are also observed, with the positive association being most evident among respondents aged 44–59, while positive coefficients are also observed in some other age groups. Gender heterogeneity is likewise apparent, as female respondents tend to report a stronger positive association between health insurance enrollment and satisfaction with healthcare facility conditions than male respondents. Regional variation is also present: the positive association is most evident among respondents in eastern China, whereas the corresponding estimates for the central and western regions are weaker and not consistently statistically significant. A similar pattern is found across household registration groups. Although both rural and urban respondents with a primary-care orientation show positive coefficients, the association appears stronger and more stable among urban residents. Overall, these findings suggest that the relationship between basic health insurance and healthcare service evaluation differs across subpopulations, but the strength and statistical significance of this association vary by subgroup and outcome dimension.

**Table 5:** Heterogeneity test of basic medical insurance evaluation of medical services for middle-aged and older adults who tend to seek primary diagnosis and treatment.

Satisfaction with healthcare facility conditions		Healthcare service level	
Variable	Regression coefficient	Variable	Regression coefficient
Chronic disease		Chronic disease	
No	0.090^*^ (0.049)	No	0.057 (0.052)
Yes	0.286 (0.173)	Yes	0.378** (0.174)
Age		Age	
44–59 years	0.157^***^ (0.061)	44–59 years	0.037 (0.064)
60–69 years	0.161^*^ (0.085)	60–69 years	0.113 (0.090)
70–79 years	−0.098 (0.126)	70–79 years	−0.105 (0.138)
80 + years	0.035 (0.278)	80 + years	−0.266 (0.343)
Gender		Gender	
Female	0.114^**^ (0.055)	Female	0.071 (0.059)
Male	0.097 (0.065)	Male	0.036 (0.068)
Region		Region	
East	0.117^**^ (0.054)	East	0.036 (0.058)
Central	0.036 (0.093)	Central	0.090 (0.096)
West	0.154 (0.094)	West	0.051 (0.100)
Household registration		Household Registration	
Non-rural household	0.210^**^ (0.101)	Non-rural Household	0.007 (0.104)
Rural household	0.094^**^ (0.047)	Rural Household	0.074 (0.051)

Note: Heteroskedasticity-robust standard errors are reported in parentheses; ***p < 0.01, **p < 0.05, *p < 0.1.

## 4 Discussion

### 4.1 Basic health insurance is associated with higher satisfaction with healthcare facility conditions

Among middle-aged and older adults with a primary-care orientation, enrollment in basic health insurance is associated with higher satisfaction with healthcare facility conditions. One possible explanation is that insurance coverage may be more directly related to respondents' perceptions of affordability, accessibility, and the organizational conditions of primary care facilities than to their evaluations of service quality itself. In the context of hierarchical diagnosis and treatment, insurance arrangements may make primary care more reachable and usable, which may in turn be reflected in more favorable evaluations of healthcare facility conditions. However, these results should be interpreted as associations rather than causal effects (41). According to the CFPS 2018 Health Section questionnaire, healthcare conditions refer to aspects such as medical services, medication, consultations, hospitalization, as well as the distance to healthcare facilities and transportation accessibility (42). Therefore, this study suggests that, under the hierarchical diagnosis and treatment system, healthcare resources are gradually being decentralized to local areas through medical consortia. Basic health insurance has played a key role in guiding middle-aged and older patients to primary healthcare facilities, may help reduce barriers related to travel distance and transportation accessibility, which directly enhances satisfaction with healthcare facility conditions. Additionally, the general practitioner system within the framework of hierarchical diagnosis and treatment, coupled with the family doctor contract system, has improved the diagnostic capabilities of primary medical institutions. This has allowed patients with common and chronic diseases to seek care at the primary level, expanding their access to healthcare services and improving satisfaction with healthcare facility conditions.

An important finding of this study is that basic medical insurance is significantly associated with satisfaction with healthcare facility conditions, but not with healthcare service level. One possible explanation is that insurance coverage may be more directly related to respondents' perceptions of the accessibility, affordability, and organizational conditions of primary care facilities than to their evaluations of service quality itself. In other words, basic medical insurance may help reduce barriers to seeking care and make primary healthcare settings appear more usable and accessible, but this does not necessarily translate into immediate improvements in perceived service level. Compared with facility conditions, healthcare service level is more likely to depend on factors such as provider competence, communication, responsiveness, and the day-to-day quality of service delivery, which may not be directly altered by insurance coverage alone. This distinction suggests that insurance expansion and service quality improvement may operate through different institutional mechanisms within the hierarchical diagnosis and treatment system.

The non-significant finding for healthcare service level should not be interpreted as evidence that insurance is unimportant. Rather, it suggests that the association between insurance coverage and healthcare evaluation may be dimension-specific. Insurance coverage appears to be more closely related to patients' perceptions of whether primary care is financially accessible and institutionally reachable, whereas evaluations of service level appear to be more closely related to provider-side factors such as professional competence, communication quality, responsiveness, and continuity of care. In this sense, insurance expansion may be a necessary but insufficient condition for improving patient-perceived service quality. This distinction is also relevant to China's hierarchical diagnosis and treatment reform: policies that expand coverage and guide patients toward primary care may improve access-related experience, but improvements in service-level evaluations are likely to require parallel investments in workforce capacity, service quality management, and primary care delivery mechanisms.

### 4.2 Heterogeneity in the association between basic health insurance and healthcare evaluation among middle-aged and older adults with a primary-care orientation

The association between basic health insurance and healthcare evaluation differs across demographic and health-related subgroups among middle-aged and older adults with a primary-care orientation. The heterogeneity analysis suggests that the association between basic health insurance and healthcare service evaluation varies across subgroups. In terms of health status, middle-aged and older adults with chronic diseases tend to report higher evaluations of healthcare service quality when enrolled in basic health insurance. This positive association is particularly evident among individuals aged 44–59, who report higher satisfaction with healthcare facility conditions than those in other age groups. Gender differences are also observed, with female respondents showing a stronger positive association between health insurance enrollment and satisfaction with healthcare facility conditions than male respondents. Regional heterogeneity is likewise apparent: respondents in eastern China report higher satisfaction with healthcare facility conditions than those in the central and western regions when covered by basic health insurance. A similar pattern is found across household registration groups. Although both rural and urban respondents with a primary-care orientation show higher satisfaction with healthcare facility conditions when enrolled in basic health insurance, the association appears to be stronger among urban residents.

These findings suggest that the association between basic health insurance and healthcare service evaluation differs across demographic groups, including health status, age, gender, region, and household registration.

## 5 Discussion

Based on these observed associations, this study offers the following policy implications for advancing the hierarchical diagnosis and treatment system.

First, the deep reform of the hierarchical diagnosis and treatment system should continue to be promoted, along with the improvement of medical insurance payment policies that support and encourage primary medical treatment. Despite the implementation of various policies in the ongoing healthcare reform, many patients still tend to choose large public hospitals for consultation (43), and issues such as “difficulty in seeing a doctor” and “high medical costs” remain unresolved (44). This study contributes by providing robust empirical evidence on the positive role basic health insurance plays in significantly enhancing patient satisfaction with primary healthcare facilities, emphasizing the need for tailored insurance payment policies that incentivize and reinforce patients' preferences for primary care.

Second, the “two-way referral” system should be further improved, emphasizing not only upward referrals but also strengthening downward referrals. The two-way referral system is the essence of the effective operation of the hierarchical diagnosis and treatment system. As China is a populous country with a large number of patients, the vulnerability of the aging population will significantly increase the pressure on healthcare services. According to the China Health and Family Planning Statistical Yearbook, the capacity of primary healthcare services has not been fully utilized. When patients are referred from primary healthcare to higher-level hospitals, a well-established system of encouraging referrals back to primary healthcare for stabilized acute and chronic disease patients should be implemented. This will alleviate medical pressures on tertiary hospitals. The findings of this research reinforce this policy recommendation by highlighting demographic groups, such as middle-aged and older adults, whose satisfaction and utilization of primary healthcare services significantly improve when supported by effective referral and insurance systems.

Third, the integration of medical information technology needs to be deepened, and the application of big data should be leveraged to promote the continuous improvement and development of the hierarchical diagnosis and treatment system. This involves the rapid establishment of electronic databases for patient medical records and health files, as well as integrating advanced technologies such as big data and artificial intelligence with traditional healthcare services through telemedicine. This technological integration can not only enhance the accuracy of disease diagnosis and the efficiency of patient management but also enable automated medical monitoring and preventive maintenance, greatly improving the effectiveness of treatment. These measures will significantly advance the hierarchical diagnosis and treatment system, particularly in providing customized and precise medical solutions. The current study further contributes to healthcare policy by underscoring how integrating these technological advancements with targeted insurance policies can foster higher patient satisfaction and increased utilization of primary medical facilities, ultimately optimizing resource allocation across healthcare tiers and advancing the efficacy of China's hierarchical diagnosis and treatment system.

## 6 Limitations

This study has several limitations. First, because the sample was restricted to middle-aged and older adults with a primary-care orientation, the findings have limited generalizability and should not be extended to all adults or to those who usually seek care in hospitals. Second, although fixed-effects models and matching strategies improve internal comparability, the observational design does not support strong causal inference. Third, the insurance variable captures enrollment status rather than the detailed generosity or benefit design of specific schemes.

The non-significant association with healthcare service level suggests that expanding insurance coverage alone may be insufficient to improve patients' perceptions of service quality, which likely requires complementary improvements in primary care capacity and service delivery. Future research should further examine whether this dimension-specific pattern varies across rural and urban settings, socioeconomic groups, and different healthcare systems, and whether such effects remain stable over time.

## 7 Conclusions

Our analysis shows that, among middle-aged and older adults with a primary-care orientation in China, basic health insurance is consistently associated with more favorable evaluations of healthcare facility conditions, whereas its association with healthcare service level is not statistically robust. These findings suggest that insurance coverage may play a meaningful role in shaping access-related experiences of primary care, but that improvements in perceived service quality likely depend on broader changes in primary care capacity and service delivery.

More broadly, our findings reiterate the need to distinguish between different dimensions of healthcare experience when evaluating the role of insurance within the hierarchical diagnosis and treatment system. By showing that insurance appears to be more strongly related to facility conditions than to perceived service level, this study adds to ongoing efforts to better understand how institutional arrangements shape patient experience in primary care. In this way, the study contributes to a more differentiated assessment of how insurance expansion and primary care reform may work together to improve healthcare delivery for aging populations in China.

## Data Availability

Publicly available datasets were analyzed in this study. This data can be found here: https://charls.pku.edu.cn/.
